# *LOXL1* gene polymorphisms are associated with exfoliation syndrome/exfoliation glaucoma risk: An updated meta-analysis

**DOI:** 10.1371/journal.pone.0250772

**Published:** 2021-04-28

**Authors:** Xiaoyan Li, Jie He, Jian Sun

**Affiliations:** 1 Department of Endocrinology, Clinical Medical College and The First Affiliated Hospital of Chengdu Medical College, Chengdu, Sichuan, China; 2 Department of Pulmonary and Critical Care Medicine, Clinical Medical College and The First Affiliated Hospital of Chengdu Medical College, Chengdu, Sichuan, China; Boston University Henry M Goldman School of Dental Medicine, UNITED STATES

## Abstract

**Background:**

Single nucleotide polymorphisms (SNPs) in the gene encoding LOXL1 are risk factors for exfoliation syndrome and exfoliation glaucoma. This meta-analysis comprehensively investigated the association between *LOXL1* gene polymorphisms (rs1048661, rs3825942, and rs2165241) and the risk of exfoliation syndrome/exfoliation glaucoma (XFS)/(XFG).

**Methods:**

All eligible case-control studies, published before August 17, 2020, were searched on Medline (Ovid), PubMed, CNKI, EMBASE, and Wanfang databases.

**Results:**

In total, 5022 cases and 8962 controls were included in this meta-analysis. Significant associations between *LOXL1* gene polymorphisms and XFS/XFG risk was observed in the disease types-based subgroups. In addition, in the subgroup analysis of ethnicity, positive associations between *LOXL1* gene polymorphisms (rs1048661, rs3825942, and rs2165241) and XFS/XFG risk were found in Caucasians. Furthermore, rs1048661 and rs3825942 polymorphisms were related to XFS/ XFG risk in Asians; however, no significant association was observed between the *LOXL1* gene rs2165241 polymorphism and XFS/XFG risk in Asians. In addition, rs1048661 and rs3825942 correlated with XFS/XFG susceptibility in Africans.

**Conclusions:**

Our results implicate *LOXL1* gene polymorphisms as XFS/XFG risk factors, especially in Caucasians.

## Introduction

Exfoliation syndrome (XFS) is an age-related, generalized disorder of the extracellular matrix characterized by progressive accumulation of abnormal fibrillar material in intra- and extra-ocular tissues [1, 2]. It is estimated to affect around 80 million people worldwide, and 10–20% of people aged >60 years are severely affected by XFS [3, 4]. This disorder is also associated with a progressive form of chronic open-angle glaucoma [2] and is the second most common cause of irreversible blindness globally.

Exfoliation glaucoma (XFG) is the most common form of secondary open-angle glaucoma and occurs in the context of XFS [4, 5]. Approximately 44% of XFS cases are estimated to progress to XFG [6]. XFG is characterized by deposition of exfoliation material in the anterior segment of the eyes, obstructing aqueous humor outflow, resulting in elevated intraocular pressure and secondary open-angle glaucoma [7]. Relative to primary open-angle glaucoma, XFS-associated secondary open-angle glaucoma is associated with a more severe prognosis, higher elevated intraocular pressure, and more severe optic nerve lesions at the time of diagnosis [8]. However, the mechanism of exfoliation material production is unclear.

XFS/XFG is a multifactorial disease involving a complex interaction between numerous risk factors, including genetic and environmental factors, myopia, cigarette smoking, and diabetes [9]. XFS/XFG prevalence varies widely across populations and geographical regions, ranging from <0.4% to >20% [10]. Thus, recent studies have increasingly focused on the relationship between gene polymorphisms and XFS/XFG susceptibility.

The lysyl oxidase-like 1 (*LOXL1*) gene has been extensively studied [11–14]. The *LOXL* family comprises five genes (*LOXL*, *LOXL1*, *LOXL2*, *LOXL3* and *LOXL4*), which encode enzymes involved in fibrillin, elastin, and collagen cross-linking reactions (2). LOXL1, which catalyzes the oxidative deamination of tropoelastin lysine residues, is essential for elastogenesis [15]. A 2007 genome-wide association study of a Scandinavian population, found a significant association between XFS/XFG and the *LOXL1* single nucleotide polymorphisms, rs1048661, rs3825942, and rs2165241, located on chromosome 15q24.1 [16]. Since then, numerous studies have affirmed that the *LOX1* polymorphisms are associated with XFS/XFG in various populations, including Caucasians, Latin Americans, Africans and Asians. Dubey et al. [17] reported that *LOXL1* G (rs1048661), G (rs3825942) and T (rs2165241) alleles are XFS/XFG risk factors in Asians, which were similar to the results found in the original study conducted by Thorleifsson et al (2007) in Caucasians (Scandinavian population), as well as in most studies carried out in Caucasians [13, 16]. However, in most studies in Asians, the alleles T and C of rs1048661 and rs2165241, respectively, are the risk alleles. Tanito et al. [18], Ozaki et al. [19], Fuse et al. [20] and Hayashi et al. [21] reported that the alleles T of rs1048661 as well as the alleles C of rs2165241 are associated with increased risk of XFS/XFG in the Japanese population. Park DY et al. [22] and Sagong et al. [11] also found a similar phenomenon in Koreans. Similar observations were made by Chen et al. in Chinese [23]. Moreover, De Juan-Marcos et al. [24] showed that the G allele of rs3825942 and the T allele of rs2165241 were XFS/XFG risk factors in a Spanish population. However, in contrast to what was observed in most Caucasian populations, no significant association between XFS/XFG and SNP rs1048661 was observed. In addition, Rautenbach et al. [25] and Williams et al. [26] indicated that the G allele of rs3825942 was protective in Black South Africans, and the G allele of rs1048661 was a risk allele for XFS/XFG. Therefore, the associations of *LOXL1* gene polymorphisms (rs1048661, rs2165241, rs3825942) may differ across patients of different ethnicities.

Despite the existence of discrepancies between some studies related to the risk alleles of *LOXL1* SNPs, it is widely accepted that *LOXL1* gene is the most important genetic risk factor known so far for XFS/XFG. Additionally, a single study may be insufficient to explore the small effect of *LOXL1* gene polymorphisms on XFS/XFG susceptibility, especially when the sample size is small. Given the associations between *LOXL1* gene polymorphisms and XFS/XFG pathogenesis, we carried out an updated meta-analysis on the correlation between *LOXL1* gene polymorphisms (rs1048661, rs2165241, rs3825942) and XFS/XFG risk. To our knowledge, this is the most comprehensive and accurate meta-analysis of *LOXL1* gene polymorphisms in the context of XFS/XFG susceptibility.

## Materials and methods

### Search strategy and criteria

Medline (Ovid), PubMed, CNKI, EMBASE, and Wanfang database searches for articles published before August 17, 2020, were performed using the following terms: “Lysyl oxidase-like 1”, “*LOXL1*”, “Exfoliation syndrome”, “XFS”, “Exfoliation glaucoma”, “XFG”, and “Polymorphism”. Articles were included if: 1) they examined the relationship between XFS/XFG susceptibility and *LOXL1* variations, 2) they were case-control studies, and 3) they had complete genotype frequency data. Articles were excluded if: 1) they lacked a control group, 2) the presented data was incomplete, 3) they were duplicate publications, and 4) controls failed to meet Hardy Weinberg Equilibrium (HWE) standards.

### Quality score evaluation

The quality of the included studies was determined using the Newcastle-Ottawa Scale [27] which assesses quality based on selection, comparability, and exposure in the study. Quality scores ranged from 0 to 9. Studies scoring >6 were considered high quality. Furthermore, study quality was determined by consensus between authors.

### Data extraction

Two independent investigators extracted tangible data from each study based on the inclusion criteria. In the case of divergent views, a third author examined the controversial articles. For each study, the first author, country, publication year, ethnicities, sample size, genotyping method, and genotype frequency in the case and control groups, were extracted.

### Statistical analyses

All analyses were conducted using STATA 10.0 and RevMan 5.2. The Odds ratio (OR) and 95% confidence interval (CI) were used to estimate the association between the *LOXL1* gene polymorphisms and XFS/XFG susceptibility. Heterogeneity among studies was evaluated using the χ^2^-based Q statistic and a *p value* ≤ 0.1 was considered statistically significant. When the *p* value was >0.1, the pooled OR of each study was calculated using a fixed-effects model. Otherwise, a random-effects model was used. The significance of the pooled OR was demonstrated using the Z-test and a *p value* ≤ 0.05 was considered statistically significant. The association between *LOXL1* gene polymorphisms and XFS/XFG risk was evaluated in different genetic models. To assess the effects of ethnicity and disease type, we performed additional subgroup analyses based on ethnicity and disease type. Sensitivity analysis was carried out to assess the stability of the results. Hardy Weinberg equilibrium was evaluated using Pearson’s χ^2^ test, and *p* ≥0.05 was considered statistically significant.

### Publication bias

Publication bias was determined using asymmetry Begger’s plots and Egger’s tests [28, 29] and was carried out using STATA 10.0.

## Results

### Study characteristics

**Fig 1:**
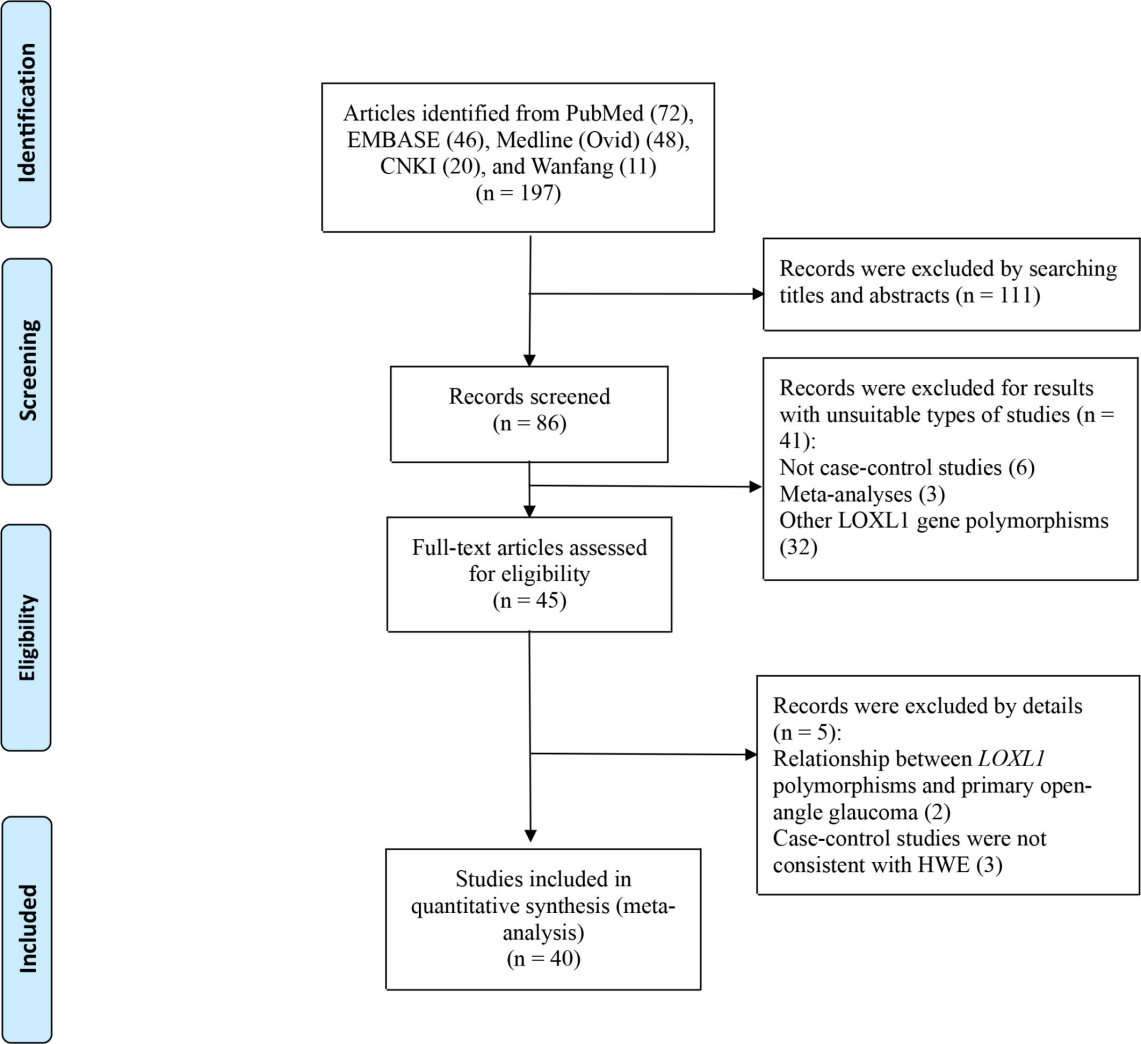
Flow diagram of studies identified.

Our initial literature search returned 197 articles. Upon browsing the titles and abstracts, 111 articles were excluded, leaving 86 articles that underwent full-text review. Of the 86 articles, 41 articles were excluded because 32 articles involved other *LOXL1* gene polymorphisms (rs4461027, rs4886761, and rsl6958477), and six articles were excluded because they were not case-control studies, and three articles were excluded for meta-analyses. Then the remaining 45 full-text articles were assessed for eligibility, although five articles [10, 30–33] had been analyzed in a previous meta-analysis [34], we excluded them because three articles [10, 30, 31] did not achieve HWE in the control group, and two articles [32, 33] reported the relationship between *LOXL1* polymorphisms and primary open-angle glaucoma. This process yielded 40 case-control articles [9, 11–14, 16–26, 35–58] that were eligible for our study (Table 1). Of these, 38 articles [9, 11–14, 16–26, 35–56] studied rs1048661, 22 articles [11, 17–20, 22–24, 35, 38–42, 45–47, 49, 52, 55–57] involved rs2165241, and 38 articles [9, 11–14, 16–26, 35–37, 39–55, 57, 58] involved rs3825942 (Fig 1).

**Table 1:** Characteristics of case-control studies included in meta-analysis on LOXL1 gene polymorphism (rs1048661, rs2165241, rs3825942).

First author	Year	Origin	Ethnicity	Type	Case	Control	Case			Control			NOS	Hardy-Weinberg equilibrium
**Ref No rs1048661**							**GG**	**GT**	**TT**	**GG**	**GT**	**TT**		
Lan [35]	2020	China	Asian	XFG	91	180	57	32	2	49	90	41	7	YES
Taghavi [36]	2019	Iran	Asian	XFS	60	40	48	12	0	24	16	0	7	YES
Pandav [37]	2018	India	Asian	XFG	30	61	17	10	3	41	16	4	6	YES
Pandav [37]	2018	India	Asian	XFS	27	61	15	10	2	41	16	4	6	YES
Shihadeh [38]	2018	Jordan	Asian	XFS/XFG	61	59	46	15	0	44	14	1	6	YES
Yaz [39]	2018	Turkey	Caucasian	XFG	58	171	46	12	0	87	64	20	6	YES
Yaz [39]	2018	Turkey	Caucasian	XFS	58	171	32	26	0	87	64	20	6	YES
Asfuroglu [40]	2017	Turkey	Caucasian	XFS	44	47	17	27	0	25	21	1	7	YES
Asfuroglu[40]	2017	Turkey	Caucasian	XFG	65	47	14	50	1	25	21	1	7	YES
De Juan-Marcos [24]	2016	Spain	Caucasian	XFS	60	90	33	25	2	47	35	8	6	YES
De Juan-Marcos [24]	2016	Spain	Caucasian	XFG	40	90	24	16	0	47	35	8	6	YES
Gayathri [13]	2016	Germany and Italy	Caucasian	XFS	48	40	26	20	2	15	20	5	8	YES
Alvarez [41]	2015	Spain	Caucasian	XFG	105	200	75	27	3	80	94	26	6	YES
Qiu [42]	2015	China	Asian	XFS	152	228	106	42	4	109	98	21	7	YES
Dubey [17]	2014	Indian	Asian	XFS	150	225	93	46	11	108	91	26	7	YES
Dubey [17]	2014	Indian	Asian	XFG	150	225	102	40	8	108	91	26	7	YES
Anastasopoulos [43]	2014	Greece	Caucasian	XFS	40	93	24	15	1	51	39	3	7	YES
Anastasopoulos [43]	2014	Greece	Caucasian	XFG	34	93	24	10	0	51	39	3	7	YES
Chiras [14]	2013	Greece	Caucasian	XFS	54	93	33	19	2	49	39	5	6	YES
Chiras [14]	2013	Greece	Caucasian	XFG	70	93	56	13	1	49	39	5	6	YES
Kasim [9]	2013	Turkey	Caucasian	XFS	100	100	77	22	1	52	38	10	6	YES
Kasim [9]	2013	Turkey	Caucasian	XFG	100	100	74	26	0	52	38	10	6	YES
Park [22]	2013	Korea	Asian	XFS/XFG	110	127	1	4	105	13	49	65	7	YES
Michael [44]	2012	Pakistan	Asian	XFG	128	180	91	36	1	78	81	21	6	YES
Rautenbach [25]	2011	South African	African	XFS	43	47	43	0	0	37	9	1	7	YES
Mayinu [45]	2011	China	Asian	XFS/XFG	64	127	42	20	2	60	56	11	7	YES
Malukiewicz [46]	2011	Poland	Caucasian	XFS	36	30	29	7	0	20	8	2	6	YES
Sagong [11]	2011	Korea	Asian	XFS	28	146	0	4	24	22	60	64	6	YES
Sagong [11]	2011	Korea	Asian	XFG	61	146	4	1	56	22	60	64	6	YES
Williams [26]	2010	South African	African	XFG	50	50	49	1	0	33	15	2	7	YES
Wolf [47]	2010	German	Caucasian	XFG	128	266	89	38	1	110	131	25	8	YES
Abu-Amero [48]	2010	Saudi Arabia	Asian	XFG	93	101	72	19	2	57	40	4	6	YES
Chen [23]	2009	China	Asian	XFS/XFG	50	125	4	3	43	23	75	27	5	YES
Lemmela [49]	2009	Finland	Caucasian	XFS/XFG	126	325	88	32	6	152	140	33	7	YES
Lee [50]	2009	China	Asian	XFS/XFG	62	171	20	25	17	29	94	48	6	YES
Ozaki [19]	2008	Japan	Asian	XFS/XFG	209	172	2	18	189	45	81	46	6	YES
Hewitt [51]	2008	America	Caucasian	XFS	86	2087	56	22	8	904	947	236	8	YES
Challa [52]	2008	America	Caucasian	XFG	47	215	29	16	2	99	88	28	7	YES
Fuse [20]	2008	Japan	Asian	XFS/XFG	56	138	1	2	53	28	80	30	7	YES
Mabuchi [53]	2008	Japan	Asian	XFS/XFG	302	191	47	108	147	40	92	59	6	YES
Mossbock [54]	2008	Australia	Caucasian	XFG	167	170	119	43	5	79	70	21	6	YES
Aragon-Martin [55]	2008	America	Caucasian	XFS/XFG	283	330	197	83	3	162	140	28	7	YES
Pasutto [56]	2008	Germany and Italy	Caucasian	XFS	280	412	179	91	10	170	194	48	7	YES
Pasutto [56]	2008	Germany and Italy	Caucasian	XFG	441	412	302	130	9	170	194	48	7	YES
Ramprasad [12]	2008	Indian	Asian	XFS/XFG	52	97	29	17	6	36	51	10	6	YES
Hayashi [21]	2008	Japan	Asian	XFS/XFG	59	189	0	1	58	37	100	52	7	YES
Tanito [18]	2008	Japan	Asian	XFS/XFG	142	251	2	10	130	65	143	43	6	YES
Thorleifsson [16]	2007	Iceland	Caucasian	XFS/XFG	128	1024	86	35	7	414	477	133	8	YES
**rs2165241**							**TT**	**TC**	**CC**	**TT**	**TC**	**CC**		
Lan [35]	2020	China	Asian	XFG	91	180	43	34	14	90	70	20	7	YES
Shihadeh [38]	2018	Jordan	Asian	XFS/XFG	61	59	38	20	3	42	12	5	7	YES
Yaz [39]	2018	Turkey	Caucasian	XFS	48	171	28	18	2	31	88	52	6	YES
Yaz [39]	2018	Turkey	Caucasian	XFG	58	171	37	18	3	31	88	52	6	YES
Asfuroglu [40]	2017	Turkey	Caucasian	XFS	44	47	21	23	0	9	31	7	7	YES
Asfuroglu [40]	2017	Turkey	Caucasian	XFG	64	47	39	25	0	9	31	7	7	YES
De Juan-Marcos [24]	2016	Spain	Caucasian	XFS	60	90	6	29	25	28	38	24	6	YES
De Juan-Marcos [24]	2016	Spain	Caucasian	XFG	40	90	2	14	24	28	38	24	6	YES
Alvarez [41]	2015	Spain	Caucasian	XFG	105	200	70	29	6	41	104	55	6	YES
Qiu [42]	2015	China	Asian	XFS	152	228	42	75	35	28	96	104	7	YES
Dubey [17]	2014	Indian	Asian	XFS	150	224	42	69	39	14	88	122	7	YES
Dubey [17]	2014	Indian	Asian	XFG	150	224	42	64	44	14	88	122	7	YES
Park [22]	2013	Korea	Asian	XFS/XFG	101	115	0	2	99	1	13	101	7	YES
Mayinu [45]	2011	China	Asian	XFS/XFG	64	127	22	28	14	10	42	75	7	YES
Malukiewicz [46]	2011	Poland	Caucasian	XFS	36	30	28	7	1	14	11	5	6	YES
Sagong [11]	2011	Korea	Asian	XFS	28	146	0	0	28	3	21	122	6	YES
Sagong [11]	2011	Korea	Asian	XFG	61	146	1	1	59	3	21	122	6	YES
Wolf [47]	2010	German	Caucasian	XFG	101	280	60	38	3	70	135	75	8	YES
Lemmela [49]	2009	Finland	Caucasian	XFS/XFG	140	316	76	53	11	65	166	85	7	YES
Chen [23]	2009	China	Asian	XFS/XFG	50	125	0	2	48	0	25	100	5	YES
Ozaki [19]	2008	Japan	Asian	XFS/XFG	209	172	2	3	204	3	29	140	6	YES
Challa [52]	2008	America	Caucasian	XFG	50	235	29	17	4	76	114	45	7	YES
Yang [57]	2008	America	Caucasian	XFS/XFG	62	170	51	9	2	49	81	40	6	YES
Tanito [18]	2008	Japan	Asian	XFS/XFG	142	251	0	2	140	5	47	199	6	YES
Fuse [20]	2008	Japan	Asian	XFS/XFG	56	138	0	2	54	0	16	122	7	YES
Aragon-Martin [55]	2008	America	Caucasian	XFS/XFG	284	328	149	119	16	60	174	94	7	YES
Pasutto [56]	2008	Germany and Italy	Caucasian	XFS	276	408	154	102	20	104	187	117	7	YES
Pasutto [56]	2008	Germany and Italy	Caucasian	XFG	441	408	272	143	26	104	187	117	7	YES
**rs3825942**							**GG**	**GA**	**AA**	**GG**	**GA**	**AA**		
Lan [35]	2020	China	Asian	XFG	91	176	76	15	0	150	23	3	7	YES
Kobakhidze [58]	2019	Georgia	Asian	XFS	132	194	99	28	5	102	62	30	7	YES
Taghavi [36]	2019	Iran	Asian	XFS	60	40	60	0	0	19	20	1	7	YES
Pandav [37]	2018	India	Asian	XFG	30	61	26	4	0	41	16	4	6	YES
Pandav [37]	2018	India	Asian	XFS	27	61	20	7	0	41	16	4	6	YES
Yaz [39]	2018	Turkey	Caucasian	XFG	58	171	58	0	0	108	57	6	6	YES
Yaz [39]	2018	Turkey	Caucasian	XFS	48	171	48	0	0	108	57	6	6	YES
Asfuroglu [40]	2017	Turkey	Caucasian	XFS	44	47	26	10	8	44	3	0	7	YES
Asfuroglu [40]	2017	Turkey	Caucasian	XFG	65	47	53	7	5	44	3	0	7	YES
De Juan-Marcos [24]	2016	Spain	Caucasian	XFS	60	90	58	1	1	66	21	3	6	YES
De Juan-Marcos [24]	2016	Spain	Caucasian	XFG	40	90	37	3	0	66	21	3	6	YES
Gayathri [13]	2016	Germany	Caucasian	XFS	48	40	45	3	0	26	9	5	8	YES
Álvarez [41]	2015	Spain	Caucasian	XFG	105	200	103	2	0	144	50	6	6	YES
Qiu [42]	2015	China	Asian	XFS	152	228	140	10	2	147	77	4	7	YES
Dubey [17]	2014	Indian	Asian	XFS	150	225	143	6	1	107	100	18	7	YES
Dubey [17]	2014	Indian	Asian	XFG	150	225	138	5	7	107	100	18	7	YES
Anastasopoulos [43]	2014	Greece	Caucasian	XFS	40	93	39	1	0	61	31	1	7	YES
Anastasopoulos [43]	2014	Greece	Caucasian	XFG	34	93	33	1	0	61	31	1	7	YES
Chiras [14]	2013	Greece	Caucasian	XFS	53	97	36	17	0	48	45	4	6	YES
Chiras [14]	2013	Greece	Caucasian	XFG	71	97	52	19	0	48	45	4	6	YES
Kasim [9]	2013	Turkey	Caucasian	XFS	100	100	100	0	0	71	26	3	6	YES
Kasim [9]	2013	Turkey	Caucasian	XFG	100	100	100	0	0	71	26	3	6	YES
Park [22]	2013	Korea	Asian	XFS/XFG	110	127	108	2	0	101	26	0	7	YES
Micheal [44]	2012	Pakistan	Asian	XFG	128	180	121	7	0	130	42	8	6	YES
Rautenbach [25]	2011	South African	African	XFS	43	47	5	2	36	19	20	8	7	YES
Mayinu [45]	2011	China	Asian	XFS/XFG	64	127	58	6	0	80	45	2	7	YES
Malukiewicz [46]	2011	Poland	Caucasian	XFS	36	30	36	0	0	23	6	1	6	YES
Sagong [11]	2011	Korea	Asian	XFS	28	146	27	1	0	116	27	3	6	YES
Sagong [11]	2011	Korea	Asian	XFG	61	146	59	2	0	116	27	3	6	YES
Williams [26]	2010	South African	African	XFG	50	50	2	9	39	20	22	8	7	YES
Wolf [47]	2010	German	Caucasian	XFG	127	272	125	2	0	196	68	8	8	YES
Abu-Amero [48]	2010	Saudi Arabia	Asian	XFG	93	101	88	4	1	70	25	6	6	YES
Chen [23]	2009	China	Asian	XFS/XFG	50	125	50	0	0	101	22	2	5	YES
Lemmela [49]	2009	Finland	Caucasian	XFS/XFG	126	325	119	6	1	224	87	14	7	YES
Lee [50]	2009	China	Asian	XFS/XFG	62	171	61	1	0	143	28	0	6	YES
Ozaki [19]	2008	Japan	Asian	XFS/XFG	209	172	205	2	2	130	37	5	6	YES
Hewitt [51]	2008	America	Caucasian	XFS	86	2089	79	5	2	1479	552	58	8	YES
Challa [52]	2008	America	Caucasian	XFG	50	235	45	5	0	177	51	7	7	YES
Yang [57]	2008	America	Caucasian	XFS/XFG	62	170	62	0	0	124	41	5	6	YES
Fuse [20]	2008	Japan	Asian	XFS/XFG	56	138	56	0	0	108	26	4	7	YES
Mabuchi [53]	2008	Japan	Asian	XFS/XFG	302	191	243	53	6	143	40	8	6	YES
Mossbock [54]	2008	Australia	Caucasian	XFG	167	170	165	2	0	109	60	1	6	YES
Aragon-Martin [55]	2008	American	Caucasian	XFS/XFG	283	332	260	23	0	216	98	18	7	YES
Ramprasad [12]	2008	Indian	Asian	XFS/XFG	52	97	45	6	1	52	40	5	6	YES
Hayashi [21]	2008	Japan	Asian	XFS/XFG	59	189	59	0	0	137	50	2	7	YES
Tanito [18]	2008	Japan	Asian	XFS/XFG	142	251	140	2	0	158	87	6	6	YES
Thorleifsson [16]	2007	Iceland	Caucasian	XFS/XFG	129	490	125	4	0	363	113	14	8	YES

### Quantitative synthesis of data

#### rs1048661 *LOXL1* gene polymorphism

**Fig 2:**
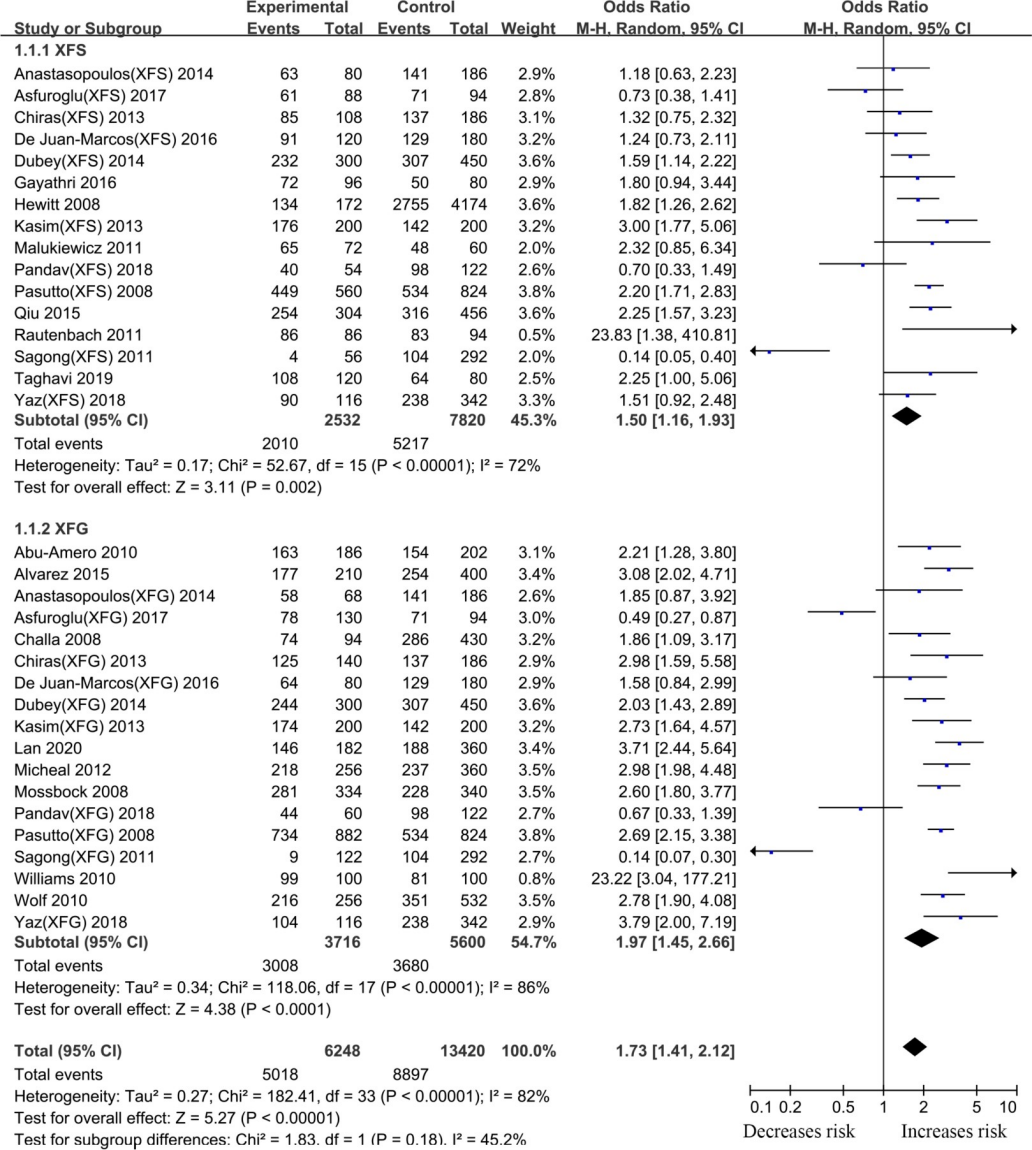
Meta-analysis for the association between exfoliation syndrome/exfoliation glaucoma risks and LOXL1 gene polymorphism rs1048661 (G vs. T): Subgroup analysis by disease types (squares depict individual studies and diamonds depict summary effect size estimates (Odds Ratio, OR)).

**Fig 3:**
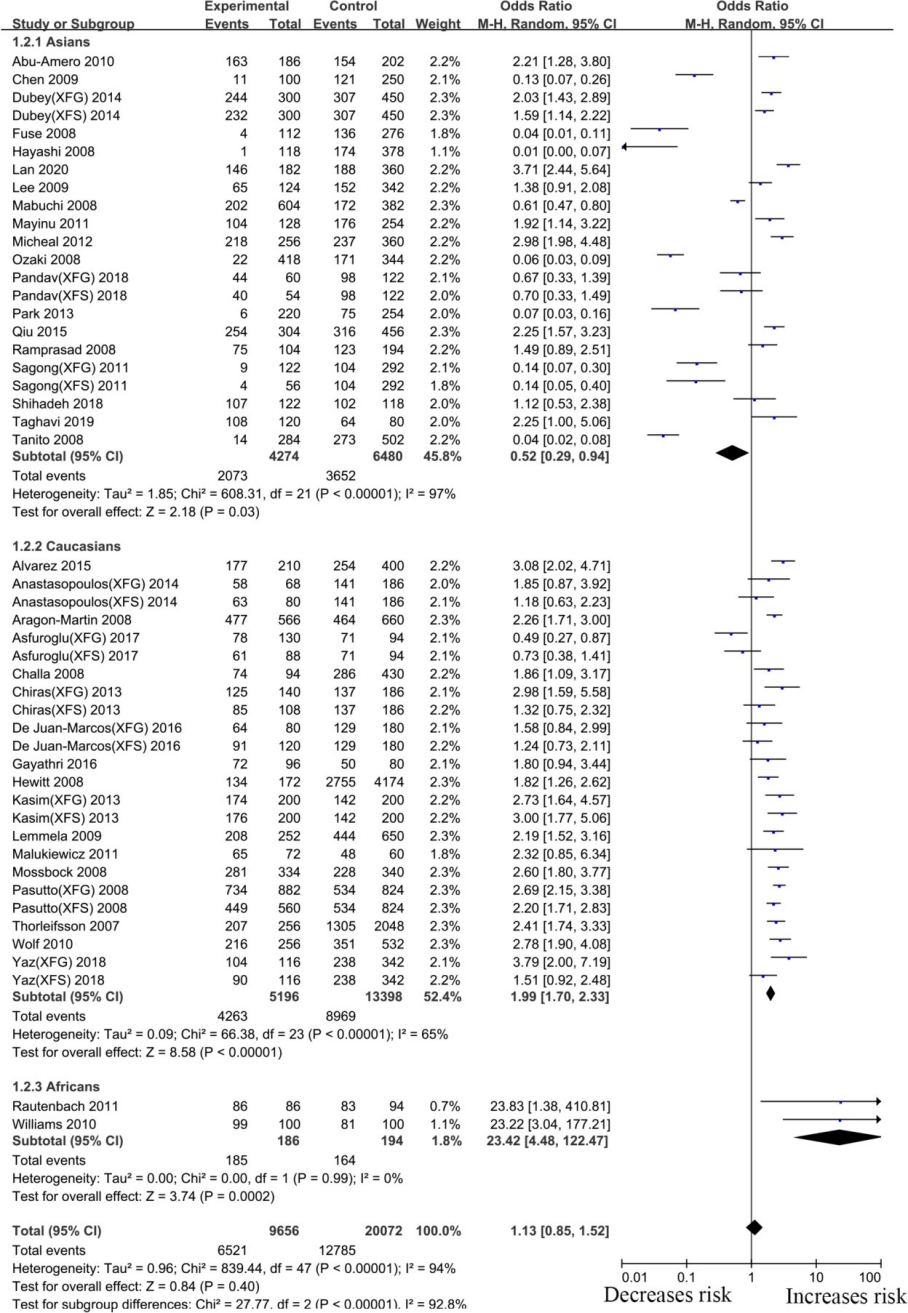
Meta-analysis for the association between exfoliation syndrome/exfoliation glaucoma risks and LOXL1 gene polymorphism rs1048661 (G vs. T): Subgroup analysis by ethnicity (squares depict individual studies and diamonds depict summary effect size estimates (Odds Ratio, OR)).

Thirty-eight articles that examined the relationship between the *LOXL1* gene polymorphism, rs1048661, and XFS/XFG risk were included in this meta-analysis. Some studies recruited XFS and XFG patients as research subjects, but these subjects did not distinguish XFS patients from XFG patients when DNA samples were sequenced. Thus, in the subgroup analysis based on the type of disease, we only extracted data from studies in which disease types (XFS or XFG) are clearly illustrated. In the subgroup analysis based on ethnicity, we combined all types of studies (XFS, XFG, XFS/XFG) to conduct the meta-analysis. Because the reason that analysis of SNPs by ethnicity is more comprehensive, we choose its merger result as the overall result. Although negative associations were found in the total sample (G vs. T, OR:1.13,95%CI: 0.85–1.52, *p*:0.40), allelic contrast analysis revealed positive associations in the XFS (G vs. T, OR: 1.50,95%CI: 1.16–1.93, *p*<0.001) and XFG (G vs. T, OR: 1.97,95%CI: 1.45–2.66, *p*<0.001) subgroups. (Fig 2, Table 2). The rs1048661 G allele was significantly correlated with higher XFG and XFS risk relative to the T allele. In the subgroup analysis of ethnicity, the meta-analysis indicated a significant association between the *LOXL1* polymorphism (rs1048661) and XFS/XFG risk in Africans (G vs. T, OR: 23.42, 95%CI: 4.48–122.47, *p* < 0.001) (Fig 3, Table 2). Notably, allelic contrast analysis showed that XFS/XFG susceptibility markedly increased in Caucasians (G vs. T, OR:1.99, 95%CI: 1.70–2.33, *p* <0.001) and significantly decreased in Asians (G vs. T, OR: 0.52, 95%CI: 0.29–0.94, *p*:0.03) (Fig 3, Table 2). In Asians, the association between rs1048661 alleles and risk was opposite to that in Caucasians and Africans. A summary of the results from other comparative genetic models is shown in Table 2.

**Table 2:** Summary of different comparative results on *LOXL1* gene polymorphism (rs1048661, rs2165241, rs3825942).

Variables/SNP	Studies	Case/Control	OR (95%CI) P	OR (95%CI) P	OR (95%CI) P	OR (95%CI) P	OR (95%CI) P
**rs1048661**			G vs. T	GT vs.TT	GG vs TT	GG+GT vs.TT	GG vs. GT+TT
Total	38	4828/10036	1.13(0.85–1.52) 0.40	0.98(0.57–1.67) 0.95	1.87(1.11–3.16) 0.019	1.4(0.78–2.54) 0.263	1.6(1.29–1.99) <0.001
Ethnicity							
Asian	19	2137/3240	0.52(0.29–0.94) 0.03	0.33(0.15–0.72) 0.005	0.54(0.23–1.27) 0.161	0.4(0.17–0.93) 0.033	0.87(0.55–1.37) 0.542
Caucasian	17	2598/6699	1.99(1.70–2.33) <0.001	2.22(1.69–2.92) <0.001	5(3.72–6.72) <0.001	3.59(2.73–4.71) <0.001	2.14(1.76–2.6) <0.001
African	2	93/97	23.42( 4.48–122.47) <0.001	0.48(0.02–15.52) 0.682	5.17(0.55–47.81) 0.148	3.89(0.42–35.81) 0.231	24.94(4.67–133.28) <0.001
Disease Category							
XFS	16	1266/3910	1.50(1.16–1.93) <0.001	1.44(1.06–1.95) 0.02	2.96(1.85–4.74) <0.001	2.1(1.18–3.73) 0.012	1.78(1.37–2.31) <0.001
XFG	18	1858/2800	1.97(1.45–2.66) <0.001	2.19(1.19–4.03) 0.012	4.81(2.41–9.6) <0.001	3.542(1.6–7.82) 0.002	2.26(1.69–3.02) <0.001
**rs2165241**			T vs. C	TC vs.CC	TT vs.CC	TT+TC vs.CC	TT vs. TC+CC
Total	22	3124/5126	1.61(1.18–2.19) 0.002	1.31(0.86–1.99) 0.207	4.47(2.59–7.7) <0.001	1.75(1.06–2.89) 0.028	2.98(.14–4.15) <0.001
Ethnicity							
Asian	11	1315/2135	0.65(0.36–1.17) 0.15	0.55(0.26–1.15) 0.112	2.11(0.93–4.79) 0.074	0.6(0.27–1.31) 0.198	1.75(0.88–3.45) 0.108
Caucasian	11	1809/2991	2.76(1.99–3.84) <0.001	2.48(1.6–3.85) <0.001	7.52(3.69–15.33) <0.001	4.34(2.32–8.14) <0.001	3.89(2.75–5.5) <0.001
Disease Category							
XFS	8	794/1344	2.14(1.33–3.45) 0.002	2.12(1.27–3.54) 0.004	4.77(1.73–13.15) 0.003	2.74(1.33–5.67) 0.006	2.82(1.55–5.11) 0.001
XFG	10	1161/1981	2(1.21–3.31) 0.007	1.67(1.67–3.15) 0.109	4.71(1.72–12.88) 0.003	2.44(1.05–5.67) 0.037	3.15(1.8–5.49) <0.001
**rs3825942**			G vs. A	GA vs. AA	GG vs. AA	GG+GA vs. AA	GG vs.GA+ AA
Total	38	4233/9017	5.33(3.49–8.16) <0.001	0.61(0.45–0.82) 0.001	2.35(1.84–2.99) <0.001	1.59(1.27–2) <0.001	6.16(4.15–9.14) <0.001
Ethnicity							
Asian	19	2208/3371	5.89(3.79–9.16) <0.001	1.04(0.66–1.63) 0.866	4.71(3.15–7.06) <0.001	3.41(2.28–5.1) <0.001	7.09(4.23–11.91) <0.001
Caucasian	17	1932/5549	6.48(3.67–11.44) <0.001	1.(0.55–1.82) 0.989	3.46(2.27–5.29) <0.001	2.88(1.87–4.43) <0.001	7.38(4.26–12.81) <0.001
African	2	93/97	0.1(0.06–0.16) <0.001	0.05(0.02–0.13) <0.001	0.04(0.01–0.1) <0.001	0.05(0.02–0.1) <0.001	0.13(0.04–0.37) <0.001
Disease Category							
XFS	17	1107/3698	4.16(1.86–9.30) <0.001	0.51(0.18–1.45) 0.206	2.61(0.96–7.1) 0.061	1.96(0.69–5.57) 0.205	5.1(2.46–10.58) <0.001
XFG	17	1420/2414	4.72(2.1–10.6) <0.001	0.49(0.22–1.13) 0.093	2.92(1.05–8.09) 0.04	2.36(0.84–6.69) 0.105	5.23(2.51–10.88) <0.001

#### rs2165241 *LOXL1* gene polymorphism

**Fig 4:**
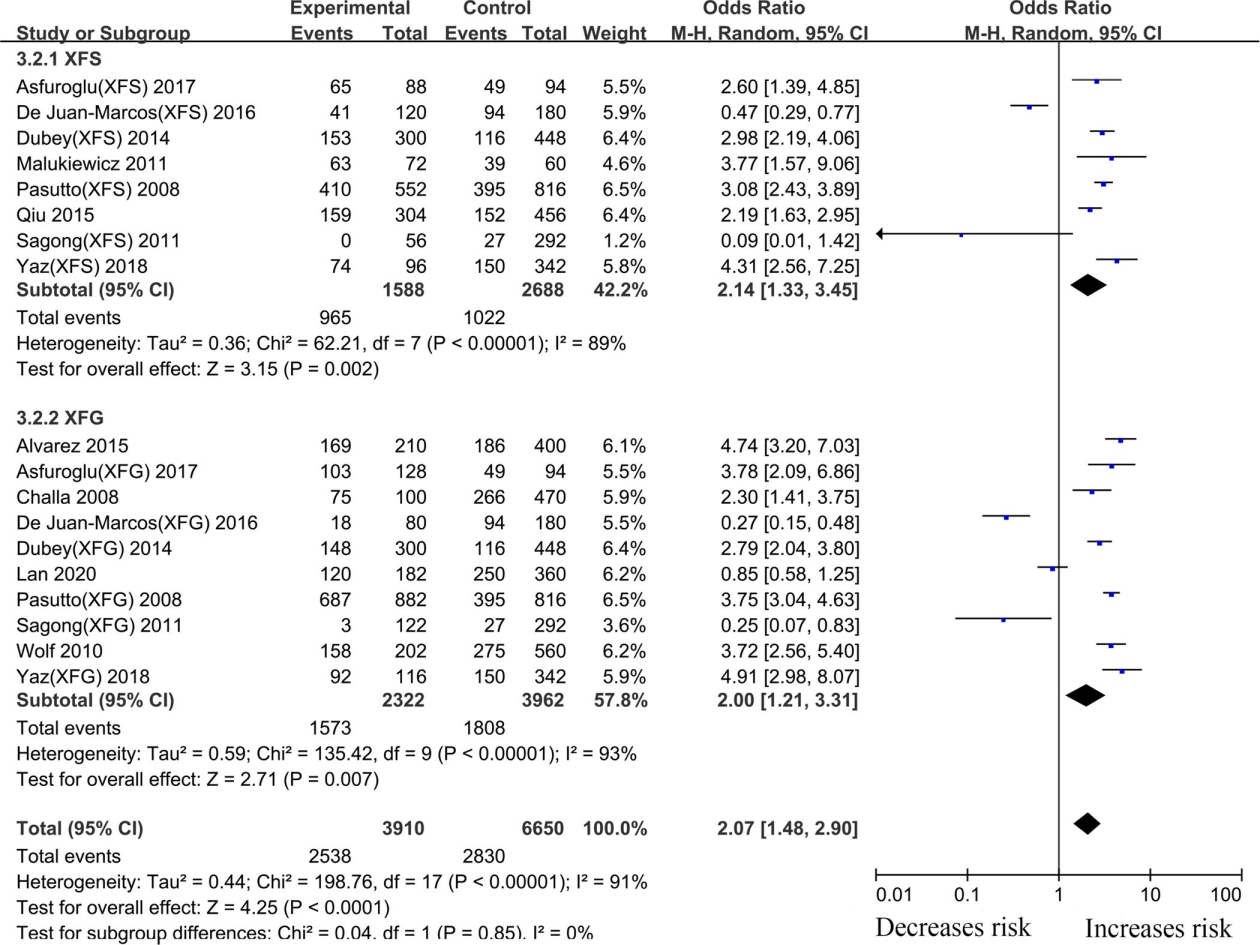
Meta-analysis for the association between exfoliation syndrome/exfoliation glaucoma risks and LOXL1 gene polymorphism rs2165241 (T vs. C): Subgroup analysis by disease types (squares depict individual studies and diamonds depict summary effect size estimates (Odds Ratio, OR)).

Twenty-two case-control articles on the relationship between the *LOXL1* gene polymorphism, rs2165241, and XFS/XFG risk were included in the meta-analysis. Overall analyses revealed a significant association between XFS/XFG susceptibility and the rs2165241 (T vs. C, OR: 1.61, 95%CI: 1.18–2.19, *p*:0.002) polymorphism (Table 2). The results revealed that genetic polymorphism of *LOXL1*(rs2165241) was associated with susceptibility to XFS (T vs. C, OR: 2.14, 95%CI: 1.33–3.45, *p*:0.002) and XFG (T vs. C, OR: 2.00, 95%CI: 1.21–3.31, *p*:0.007) (Fig 4, Table 2), in the allelic contrast.

**Fig 5:**
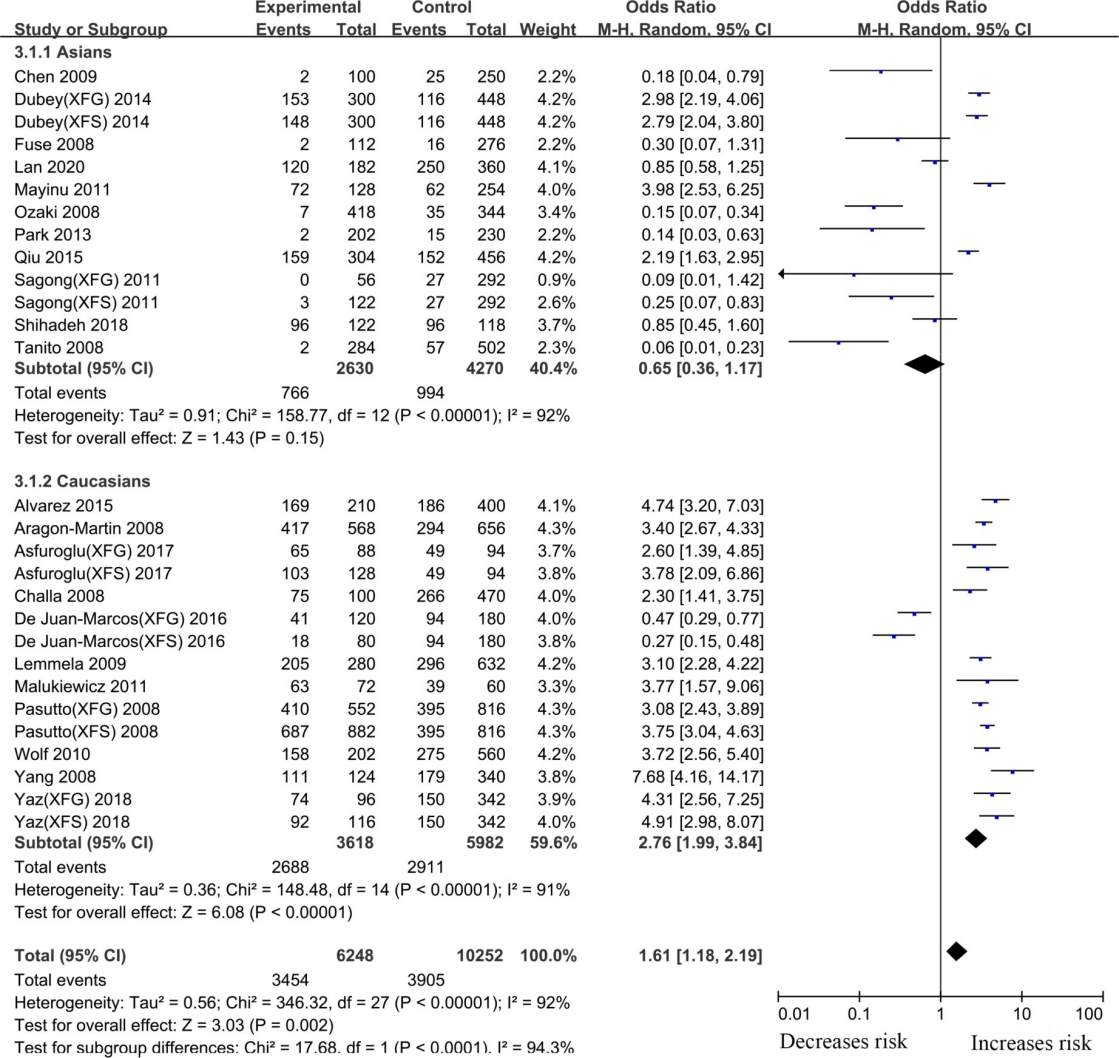
Meta-analysis for the association between exfoliation syndrome/exfoliation glaucoma risks and LOXL1 gene polymorphism rs2165241 (G vs. A): Subgroup analysis by ethnicity (squares depict individual studies and diamonds depict summary effect size estimates (Odds Ratio, OR)).

Subgroup analysis by ethnicity identified an increased risk in Caucasians (T vs. C, OR: 2.76, 95%CI: 1.99–3.84, *p* <0.001) (Fig 5, Table 2). However, there was no significant association between the *LOXL1* gene rs2165241 polymorphism and XFS/XFG risk in Asians (T vs. C, OR: 0.65, 95%CI: 0.36–1.17, *p*:0.15) (Fig 5, Table 2). A summary of the results from other comparative genetic models is also shown in Table 2.

#### rs3825942 *LOXL1* gene polymorphism

**Fig 6:**
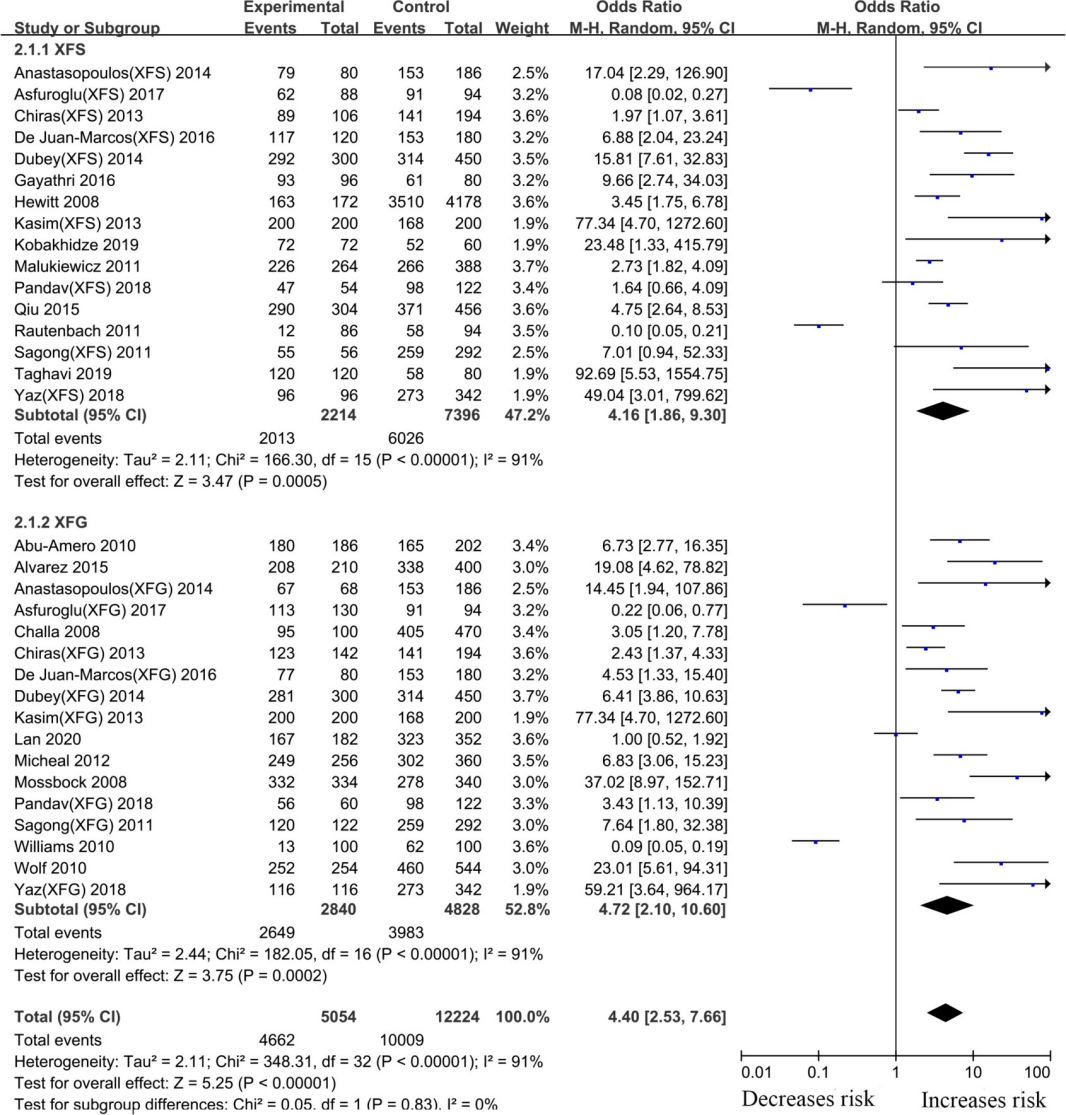
Meta-analysis for the association between exfoliation syndrome/exfoliation glaucoma risks and LOXL1 gene polymorphism rs3825942 (G vs. A): Subgroup analysis by disease types (squares depict individual studies and diamonds depict summary effect size estimates (Odds Ratio, OR)).

**Fig 7:**
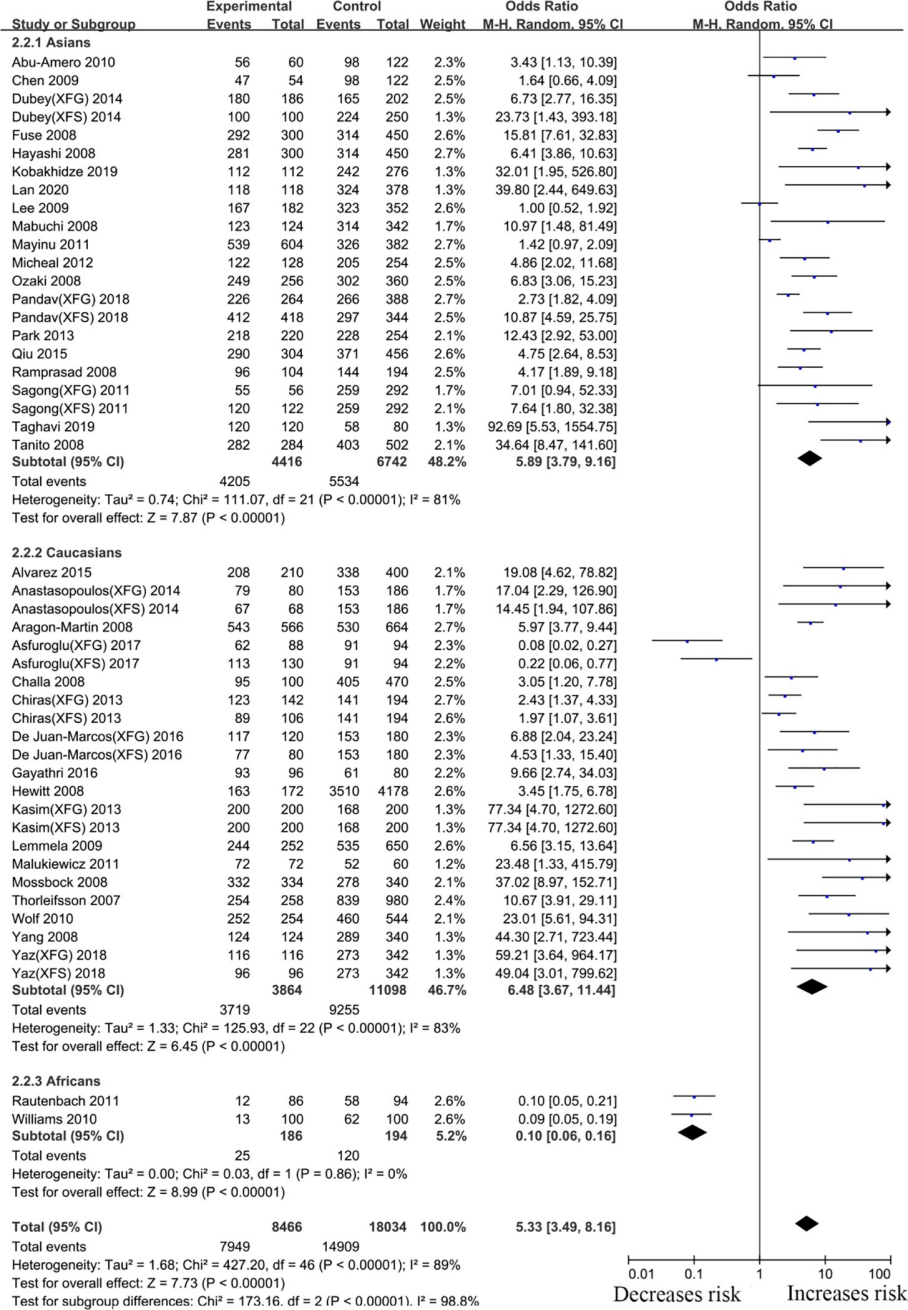
Meta-analysis for the association between exfoliation syndrome/exfoliation glaucoma risks and LOXL1 gene polymorphism rs3825942 (G vs. A): Subgroup analysis by ethnicity (squares depict individual studies and diamonds depict summary effect size estimates (Odds Ratio, OR)).

For the *LOXL1* gene polymorphism, rs3825942, 38 articles were included in our meta-analysis. Overall analyses revealed a positive *LOXL1* rs3825942 (G vs. A, OR: 5.33, 95%CI: 3.49–8.16, *p* <0.001) association with XFS/XFG susceptibility (Table 2). In the subgroup analysis by disease type, the *LOXL1* rs3825942 gene polymorphism revealed a significant association with XFS (G vs. A, OR: 4.16, 95%CI: 1.86–9.30, *p* <0.001) and XFG (G vs. A, OR: 4.72, 95%CI: 2.10–10.60, *P*<0.001) (Fig 6, Table 2) susceptibility in a genetic model, G vs. A. In subgroup analysis by ethnicity, increased risks were identified among Caucasians (G vs. A, OR: 6.48, 95%CI: 3.67–11.44, *P*<0.001) and Asians (G vs. A, OR: 5.89, 95%CI: 3.79–9.16, *p* <0.001) (Fig 7, Table 2), suggesting that variant G allele carriers are at higher risk of XFS/XFG relative to A allele carriers. In contrast, the G allele indicates protection from XFS/XFG in Africans (G vs. A, OR: 0.10, 95%CI: 0.06–0.16, *p* <0.001). A summary of the results from other comparative genetic models is shown in Table 2.

### Publication bias and sensitivity analyses

**Fig 8:**
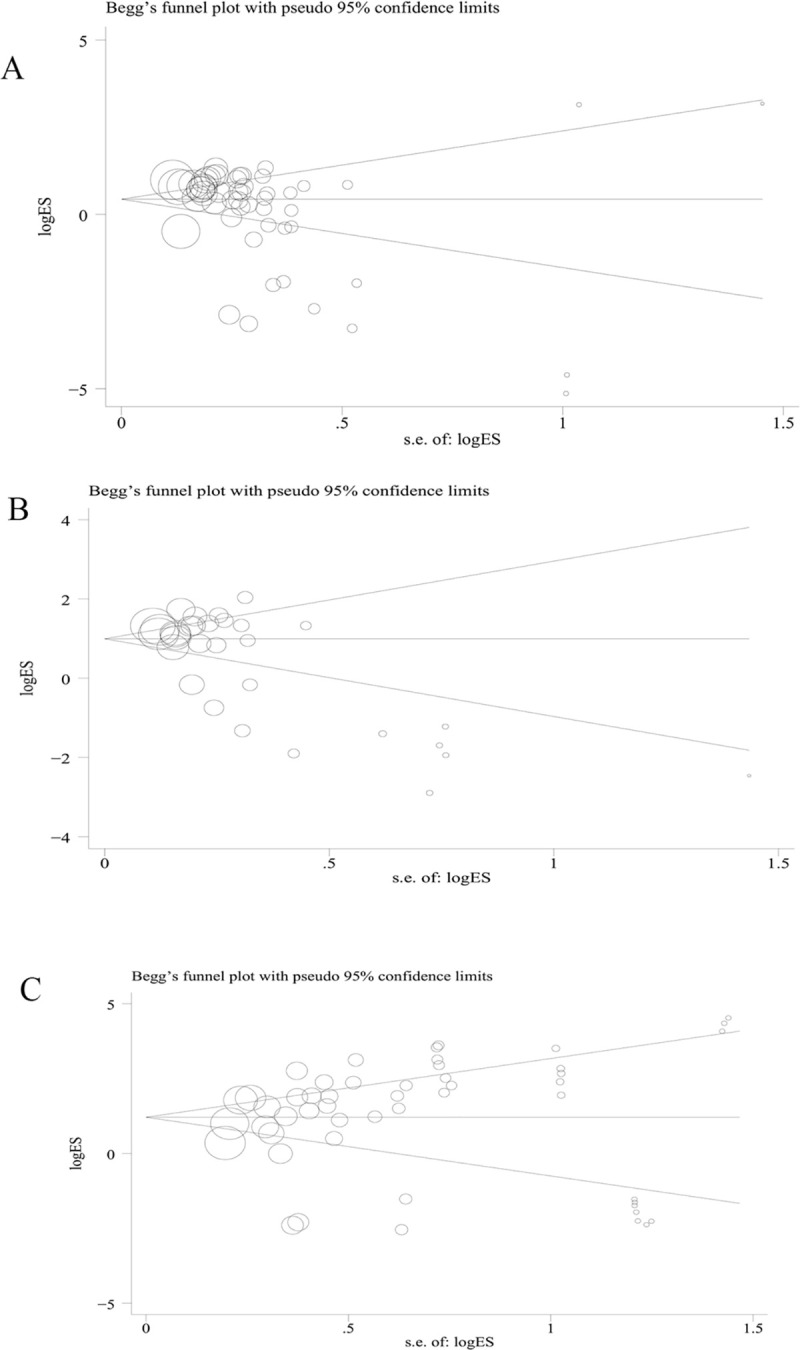
(A) Begg’s funnel plot of publication bias for LOXL1 gene polymorphism rs1048661; (B) Begg’s funnel plot of publication bias for LOXL1 gene polymorphism rs2165241; (C) Begg’s funnel plot for of publication bias for LOXL1 gene polymorphism rs3825942.

Funnel plot pictures were symmetrical inverted funnels. Egger’s test was used to provide statistical evidence of the funnel plot (rs1048661: *t* = 1.62, *p* = 0.114; rs3825942: *t* = 1.26, *p* = 0.215; rs2165241: *t* = -2.10, *p* = 0.148) (Fig 8). To determine the potential source of heterogeneity, we performed a sensitivity analysis by sequentially excluding studies from the meta-analysis and assessing the effect of each article on the pooled results. This analysis did not reveal any significant alterations to the pooled ORs, indicating the stability of the three polymorphisms studied.

## Discussion

XFS is associated with a high morbidity and blindness rate [5]. This systemic disease of the extracellular matrix, may cause pathological material accumulation in blood vessels, skin, heart, lung, liver, and cerebral meninges [59]. XFG, which results from XFS, is the most common identifiable cause of secondary open-angle glaucoma and is associated with cataracts [60–62]. Additionally, XFG increases the risk of potentially sight-threatening conditions and serious complications from cataract surgery [59]. Numerous studies indicate that XFS/XFG risk factors include inflammation, immune dysfunction, oxidative stress, unhealthy lifestyle, and various environmental factors [63]. Owing to clustering of XFS/XFG in families, concordance in monozygotic twins, and prevalence variability by ethnicity, genetic factors are regarded as XFS/XFG risk factors [64–68]. It is widely accepted that the *LOXL1* gene is the most important genetic risk factor known so far for XFS/XFG. Besides, a single study might lack sufficient power to detect the potential small *LOXL1* gene polymorphism effects associated with XFS/XFG, especially when the sample size is not adequate. Thus, a meta-analysis may effectively identify the association between genetic risk factors and XFS/XFG as such quantitative analyses integrate results from numerous studies on the topic of study, potentially drawing more objective and reliable conclusions. Here, we conducted a pooled analysis to evaluate the association between *LOXL1* gene polymorphisms and XFS/XFG susceptibility.

Three recent meta-analyses [34, 69, 70] investigated the association between the *LOXL1* gene polymorphisms and XFS/XFG risk. However, all of them covered papers published until to 2015, with the latest data unrepresented. Tang C et al. [69] and Chen H et al. [70] both indicated that the allele G of rs1048661, the allele T of rs2165241 and the allele G of rs3825942 were associated with an increased risk for XFS/XFG among Caucasians, and that only the allele G of rs1048661 and the allele T of rs2165241 had a potential protective effect on XFS/XFG in Asians. Nevertheless, our study showed that there was no significant association between the LOXL1 gene rs2165241 polymorphism and XFS/XFG risk in Asians, and that rs3825942 (“G” allele) carriers are at higher risk of XFS/XFG relative to A allele carriers in Asians. On this point, our conclusion seems partially inconsistent with the previous two meta-analyses. Moreover, their study did not involve XFS/XFG in Africans, which is important and worthy of attention. Wang L et al. [34] reported that rs1048661(“G” alleles) had a weak association with XFG/XGS; rs3825942 (“G” alleles) had a strong association with XFS/XFG; and rs2165241 (“T” alleles) had a significant risk with XFS/XFG in Caucasians. Our meta-analysis has corroborated their findings. However, three articles [10, 30, 31] included in Wang’s meta-analysis [34], did not achieve HWE in the control group, while two articles [32, 33] examined the relationship between *LOXL1* gene polymorphisms and primary open-angle glaucoma. Here, we carried out an updated meta-analysis of the association between *LOXL1* gene polymorphisms and XFS/XFG susceptibility, involving 13984 participants. We identified three polymorphisms, rs1048661, rs3825942, and rs2165241, that met the inclusion criteria for meta-analysis. XFS/XFG analysis by ethnicity revealed a significantly high association between the G allele of rs1048661, the allele T of rs2165241 and the allele G of rs3825942, and XFS/XFG risk in Caucasians. We found that the G allele of rs1048661 may have potentially negative effects on XFS/XFG in Africans, and the G allele of rs3825942 may protect from XFS/XFG in Africans. In Asians, a significantly increased XFS/XFG risk was associated with the G allele of rs3825942. However, we also found that the G allele of rs1048661 was associated with reduced XFS/XFG risk in Asians. In Asians, there was no significant association between the T allele of rs2165241 and XFS/XFG risk. Additionally, there was a significant association between *LOXL1* gene polymorphisms and susceptibility to various disease types. These results affirmed the association between *LOXL1* gene polymorphisms and XFS and XFG. Notably, we found a high frequency of risk alleles (rs1048661, rs2165241, and rs3825942) in non-XFG/XFS individuals, especially in Caucasians. Some studies have reported that these polymorphisms affect the proteolytic activity of LOXL1, and LOXL1 is an important matrix cross-linking enzyme that is required for elastic fiber formation and confer risk for the development of XFS/XFG [71]. However, the contribution of the risk alleles to XFS/XFG is complicated. Certain genetic variants of LOXL1, which has a prominent role in elastin fiber production, are not a single causative factor as many genetically affected individuals do not develop XFS or XFG [72]. It is likely that additional genetic or environmental factors modulate the penetrance of *LOXL1* susceptibility alleles [52]. This meta-analysis found that *LOXL1* gene polymorphisms may contribute to XFS/XFG susceptibility in different populations, and the differences in genetic susceptibility might be affected by ethnic factors, lifestyle factors and environmental exposures. Unfortunately, there are few studies concerning the association between *LOXL1* gene polymorphisms (rs3825942 and 1048661) and XFS/XFG in Africans, and no data were available for the SNP rs2165241 in Africans. This may lead to bias in the conclusion and generalization of the relationship between *LOXL1* gene polymorphisms and XFS/XFG in Africans. Thus, many such original studies are needed to confirm these findings as the currently included case-control studies are based on small sample sizes, especially for African populations.

The mechanisms by which *LOXL1* gene polymorphisms affect XFS/XFG susceptibility remain unclear. Multiple studies [63, 73] have shown that LOXL1 mediates the formation and maintenance of elastic tissues, as well as maintenance of extracellular matrix homeostasis, by regulating cross-linking reactions between collagen and elastin. LOXL1 has also been reported to be involved in elastin renewal and XFS/XFG development [71, 74]. Sharma et al. [59] reported that the coding variants rs1048861 and rs3825942 may alter protein function and binding, wherein molecular modeling displayed that positions 141(rs1048661) and 153(rs3825942) of the LOXL1 protein are likely surface residues and hence possible recognition sites for protein-protein interactions. Alterations at these residues might change the capacity of LOXL1 to bind other proteins related to its cleavage as well as processing. Nevertheless, the difference in processing of the LOXL1 protein variants detected in their research does not completely interpret susceptibility to XFS/XFG among carriers of these variants as each of the variants confer the XFS/XFG risk in various ethnicities. The detailed mechanism whereby *LOXL1* gene polymorphisms lead to the XFS/XFG, remains poorly understood. Therefore, further studies are required to elucidate the mechanism on how the *LOXL1* gene polymorphisms impact the occurrence and development of XFS/XFG. Moreover, the distinct genetic background of Caucasians from Asians may modify LOXL1-mediated genetic susceptibility; hence, the effects of rs2165241 and rs1048661 are opposite in Asians and Caucasians. Genetic and/or environmental factors may modify the effects of gene polymorphisms in different ethnic groups.

High heterogeneity was found in our study. For exploring the underlying source of heterogeneity, a subgroup analysis and sensitivity analysis were performed. Unfortunately, although subgroup and sensitivity analyses were performed, obvious heterogeneity still existed in certain genetic models, and it is difficult to explain the heterogeneity completely. Thus, we speculated that living environment and other complications might lead to heterogeneity. Publication bias was assessed using Begg’s funnel plot and Egger’s test; no significant publication bias was found in this meta-analysis. Moreover, all genotype distributions of controls were in -absolute accordance with the HWE, indicating that our results are stable and reliable.

We acknowledge several limitations of this study. First, in subgroup analysis by ethnicity and disease type, some subgroups consisted of less than three case-control studies, which may be too small to detect associations. Second, data were not stratified by other factors, such as gender, age, gene-environment/gene-gene interactions, and lifestyle, because sufficient information could not be extracted from primary publications. Third, we mainly focused on *LOXL1* gene polymorphisms, and did not take into consideration potential linkage disequilibrium with other mutations in this gene, or gene-gene and gene-environment interactions. Moreover, language bias may have occurred as only articles published in Chinese or English were included in the study. However, we minimized the likelihood of bias using a rigorous protocol, study identification, data selection, and statistical analysis.

## Conclusion

In conclusion, our findings indicate that rs1048661, rs3825942, and rs2165241 *LOXL1* polymorphisms may contribute to XFS/XFG susceptibility, especially in Caucasians. Furthermore, well-designed studies with large sample sizes focusing on ethnicity or disease types are needed to confirm these findings.

## Supporting information

S1 File PRISMA 2009 checklist. (DOC)
