# Supplementary material for: LOXL1 gene polymorphisms are associated with exfoliation syndrome/exfoliation glaucoma risk: An updated meta-analysis
Source: PLoS One. 2021 Apr 28;16(4):e0250772. doi: 10.1371/journal.pone.0250772 (PMC8081202; doi:10.1371/journal.pone.0250772)
Supplement: Attachment — Submitted filename: Response to Reviewers.doc [file pone.0250772.s002.doc]

**Reply:** The authors would like to thank the Reviewers and the Editor for their time in evaluating our study and for making useful suggestions. We believe that we have addressed all of the comments below, but we are open to any additional ones.

**Reply to Editors：**

1. Please ensure that your manuscript meets PLOS ONE's style requirements, including those for file naming.

**Reply:** We have revised the manuscript to meet PLOS ONE's style requirements.

2. Please include your tables as part of your main manuscript and remove the individual files. Please note that supplementary tables should be uploaded as separate "supporting information" files.

**Reply:** We have included the tables in my main manuscript and removed the individual files.

3. Thank you for stating the following financial disclosure: "none"

At this time, please address the following queries:

1. Please clarify the sources of funding (financial or material support) for your study. List the grants or organizations that supported your study, including funding received from your institution.
2. State what role the funders took in the study. If the funders had no role in your study, please state: “The funders had no role in study design, data collection and analysis, decision to publish, or preparation of the manuscript.”
3. If any authors received a salary from any of your funders, please state which authors and which funders.
4. If you did not receive any funding for this study, please state: “The authors received no specific funding for this work.”

**Reply:** We have added the state in manuscript in Page 21 in manuscript.

4.  Thank you for stating the following in your Competing Interests section:

"none"

Please complete your Competing Interests on the online submission form to state any Competing Interests. If you have no competing interests, please state "The authors have declared that no competing interests exist.", as detailed online in our guide for authors at http://journals.plos.org/plosone/s/submit-now

 This information should be included in your cover letter; we will change the online submission form on your behalf.

**Reply:** We have added the state in manuscript in Page 21 in manuscript and cover letter.

5. In your Data Availability statement, you have not specified where the minimal data set underlying the results described in your manuscript can be found. PLOS defines a study's minimal data set as the underlying data used to reach the conclusions drawn in the manuscript and any additional data required to replicate the reported study findings in their entirety. All PLOS journals require that the minimal data set be made fully available. For more information about our data policy, please see <http://journals.plos.org/plosone/s/data-availability.>

Upon re-submitting your revised manuscript, please upload your study’s minimal underlying data set as either Supporting Information files or to a stable, public repository and include the relevant URLs, DOIs, or accession numbers within your revised cover letter. For a list of acceptable repositories, please see [http://journals.plos.org/plosone/s/data-availability#loc-recommended-repositories.](http://journals.plos.org/plosone/s/data-availability#loc-recommended-repositories.) Any potentially identifying patient information must be fully anonymized.

**Reply:** All relevant data are within the paper and its Supporting information files.

7. Please include captions for your Supporting Information files at the end of your manuscript, and update any in-text citations to match accordingly.

 Reply: All relevant Supporting Information files are within the paper and updated the citations one by one.

**Reply to Reviewers：**

**Reply:** We thank the reviewers’ comments and excellent suggestions. We have revised these mistakes and inaccuracies in the text as you pointed out, rewritten some references and revised some parts of the text. In addition, we have consulted a professional company Editage for proofreading to polished the language in the manuscript. Reply to comments and suggestions of reviewers as follows:

1. Human gene symbols must be italicized throughout the text. LOXL1 when you refer to the gene. LOXL1 for the protein.

**Reply:** We have italicized throughout the text of the LOXL1 gene.
2. Page 7, Abstract background, first sentence: “Lysyl oxidase-like 1 (LOXL1), a matrix cross-linking enzyme involved in elastic fiber formation, is a risk factor for exfoliation syndrome and exfoliation glaucoma” This sentence should be corrected.
Single nucleotide polymorphisms (SNPs) in the gene encoding the LOXL1 are the risk factors for exfoliation syndrome and exfoliation glaucoma”.

**Reply:**We have revised the sentence in Page 1 in manuscript.
3. Page 8, Abstract results: “… was observed in the disease types-based subgroup” should be “were observed in the disease type-based subgroups”

**Reply:**We have revised the sentence in Page 2 in manuscript.
4. Page 8, Abstract results: please clarify “…combined polymorphism…”

**Reply:** We have replaced the words to a more proper sentence in Page 2 in manuscript.
5. Page 8, Abstract results: “relative to” should be “related to”

**Reply:** We have revised the words in Page 2 in manuscript.
6. Page 8, Introduction, paragraph 1: please clarify “occoecatio”. Is there another better known term with the same meaning?

**Reply:** We have replace the word to “blindness” in Page 2 in manuscript.
7. Page 8, Introduction, paragraph 1: Reference 4 is before reference 3 in the text.

**Reply:** We have removed the reference 4 to ahead of the former 3 in Page 2 in manuscript.
8. The article corresponding to reference number 3 in the list of references, has been retracted by the editor of International Journal of Ophthalmology. This reference should be removed from the article.

**Reply:** We have removed the reference in the article in Page 2 in manuscript.
9. Page 9, paragraph 1: “intraocular pressure” should be “elevated intraocular pressure”

**Reply:** We have revised the sentence in Page 3 in manuscript.
10. Page 9, paragraph 1: Add a comma “Relative to primary open-angle glaucoma, XFS-associated secondary open-angle glaucoma...”

**Reply:** We have added a comma in the sentence in Page 3 in manuscript.
11. Page 9, paragraph 3: reference 16 is wrong. The correct reference is: Thorleifsson G, Magnusson KP, Sulem P, Walters GB, Gudbjartsson DF, Stefansson H et al. Common sequence variants in the LOXL1 gene confer susceptibility to exfoliation glaucoma. Science. 2007;317(5843):1397-1400.

**Reply:** We have revised the reference, and revised the number of the following reference.
12. Page 9, paragraph 3: This statement is incorrect: “Dubey SK et al (17) reported that LOXL1 G (rs1048661) and T (rs3825942) alleles are XFS/XFG risk factors in Caucasians.” The work of Dubey et al was carried out in an Asian population no in Caucasians. Moreover, there is also some mistake regarding the risk alleles.
Dubey et al reported that the G allele of rs1048661, the G allele of rs3825942 and the T allele of rs2165241 were XFS/XFG risk factors in a South Indian population. Which is similar to that found in the original study conducted by Thorleifsson et al (2007) in Caucasians (Scandinavian population), as well as in most studies carried out in Caucasians, but different to that reported in most studies in Asians, where the alleles T and C of rs1048661 and rs2165241, respectively, are the risk alleles. References of these works should be added.

**Reply:** This statement is incorrect: “Dubey SK et al. reported that LOXL1 G (rs1048661) and T (rs3825942) alleles are XFS/XFG risk factors in Caucasians.” Has been re-written to “Dubey SK et al. reported that LOXL1 G (rs1048661), G (rs3825942) and T (rs2165241) alleles are XFS/XFG risk factors in Asians. Dubey SK et al' results is similar to that found in the original study conducted by Thorleifsson et al (2007) in Caucasians (Scandinavian population), as well as in most studies carried out in Caucasians” in Page 4 in manuscript.
13. Pages 9-10, Paragraph 3 of page 9 and 1 of page 10: The sentence “De Juan-Marcos L et al (18) showed that LOXL1 rs2165241 polymorphism may be associated with XFS/XFG susceptibility” should be corrected.
De Juan-Marcos et al showed that the G allele of rs3825942 and the T allele of rs2165241 were XFS/XFG risk factors in a Spanish population. But no significant association between XFS/XFG and the SNP rs1048661 was observed. This last observation is the most relevant, since it shows a discrepancy with the majority of studies in Caucasian populations. References of these works should be added.

**Reply:** It has been corrected to“Moreover, De Juan-Marcos et al showed that the G allele of rs3825942 and the T allele of rs2165241 were XFS/XFG risk factors in a Spanish population. But no significant association between XFS/XFG and the SNP rs1048661 was observed” in Page 4 in manuscript.
14. Page 10, paragraph 1: The statement “However, Tanito M et al (19) reported that LOXL1 G and T alleles are protective against XFS/XFG in Japanese populations. Similar observations were made by Chen L et al (20)” should be extended to more Asian studies, where G (rs1048661) and T (rs2165241) alleles are protective against XFS/XFG, and not only to the work of Chen L et al (20) in a Chinese population. References of these works should be added. See point 12.

**Reply:** It has been corrected to “However, different to that reported in most studies in Asians, where the alleles T and C of rs1048661 and rs2165241, respectively, are the risk alleles. Tanito M et al., Ozaki, Fuse, Hayashi, reported that the alleles T of rs1048661 as well as the alleles C of rs2165241 is associated with increased risks of XFS/XFG in Japanese . Park DY et al., Sagong, found a similar phenomenon in Koreans. Similar observations were made by Chen L et al in Chinese” in Page 4 in manuscript.
15. Page 10, paragraph 1: Some reference should be made to the results found in the literature for the black race and discrepancies with other ethnicities.

**Reply:** It has been corrected to “In addition, Rautenbach et al. and Williams et al. both indicated that the G allele of rs3825942 was protective in Black South Africans, and the G allele of rs1048661 was at risk allele for XFS/XFG” in Page 4 in manuscript.
16. Page 10, paragraph 2: The statement “Past genetic predisposition studies have been inconclusive” should be qualified.
Despite the existence of discrepancies between some studies related with the risk alleles of LOXL1 SNPs, it is widely accepted that LOXL1 gene is the most important genetic risk factor known so far for XFS/XFG.

**Reply:** We have revised the sentence of the state in Page 4 in manuscript.
17. Page 10, Material and methods: Please clarify the point “3) if they consisted of a complete number of genotypes among cases and controls”

**Reply:** We have revised the sentence to “they had complete genotype frequency data” in Page 5 in manuscript.
18. Page 11, Statistical analyses: Please, replace “gene models” by “genetic models”

**Reply:** We have revised the sentence in Page 6 in manuscript.
19. Page 11, Statistical analyses: Add software/s used to carry out the statistical analyses.

**Reply:** We have added the software about statistical analysis in Page 6 in manuscript..
20. Page 12, Publication bias: References 22 and 23 are not the most suitable. I suggest the following references:
Peters JL, Sutton AJ, Jones DR, et al. Contour-enhanced meta-analysis funnel plots help distinguish publication bias from other causes of asymmetry. J Clin Epidemiol 2008;61:991–6.
Sterne J A, Gavaghan D, Egger M. Publication and related bias in meta-analysis: power of statistical tests and prevalence in the literature. J Clin Epidemiol 2000;53:1119–29.

**Reply:** We have revised the two references as you recommended.
21. Page 12, 3.1 Study characteristics: Please, correct Figure 1. According to the text, titles and abstracts where searched in step 1 and not in step 2 as showed in the Figure 1.

**Reply:** We have revised the Figure 1 according to the text in Page 6 in manuscript.
22. Page 12, 3.1 Study characteristics, line 6, “a previous meta-analysis” should be “in a previous meta-analysis”

**Reply:** We have revised the sentence in Page 7 in manuscript.
23. Table 2: Please, specify what is N

**Reply:** We have replaced the N to Studies in Page 7 in manuscript.
24. I suppose that N is the number of studies, but in table 2, for rs1048661, N is 39 instead of 38 as showed in the text (page 13, line 1).

**Reply:** We have checked the numbers that the studies of rs1048661 is 38 indeed in Page 12 in manuscript.
25. In Table 2 N is 17 for rs1048661, XFS, however, in Figure 2 only 16 studies are listed.

**Reply:** We have revised the N in Table 2 in Page 7 in manuscript.
26. In Table 2 N is 21 for rs1048661 in Asians, however, in Figure 3 only 19 different studies are listed.

**Reply:** We have revised the N in Table 2 in Page 7 in manuscript.
27. In Table 2 N is 18 for rs1048661 in Caucasians, however, in Figure 3 only 17 different studies are listed.

**Reply:** We have revised the N in Table 2 in Page 7 in manuscript.
28. Please, clarify y/o correct the following discrepancies between the data of the tables 1 and 2:
- In table 2 the total number of cases (XFS/XFG) is 4900 for rs1048661, however, if the number of cases from each study listed in Table 1 are added, the total number of cases is 4828.
- In table 2 the total number of controls (XFS/XFG) is 10111 for rs1048661, however, if the number of controls from each study listed in Table 1 are added, the total number of cases is 10036.
- In table 2 the total number of cases (XFS/XFG) is 4305 for rs3825942, however, if the number of cases from each study listed in Table 1 are added, the total number of cases is 4233.
- In table 2 the total number of controls (XFS/XFG) is 9092 for rs3825942, however, if the number of controls from each study listed in Table 1 are added, the total number of cases is 9017.

**Reply:** We have revised the N in Table 2 in Page 7 in manuscript.
29. Page 13, first paragraph: The authors claim that no associations were found between the SNP rs1048661 and XFS/XFG (total group). That is according to table 1 and Fig3 data but however, according to the data of figure 2, there is association. OR=1.73-1.41, 2.12.
Please, explain more in detail the two analysis realized for each SNP (by disease and by ethinicity) and why analysis considering ethnicity is chosen.
It should be clarify in the text that the data “G vs. T, OR:1.13,95%CI: 0.85-1.52, P:0.40” are shown in Fig3.

**Reply:** We have clarified the reason of the discrepancy is that “Some studies included patients with XFS and XFG, but did not distinguish which were XFS patients, and which were XFG patients. Thus, in the subgroup analysis based on the type of disease, we only extracted data from studies in which disease types (XFS or XFG) are clearly illustrated. While, in the subgroup analysis based on ethnicity, we combine all types of studies (XFS, XFG, XFS/XFG) to conduct a meta-analysis. For the reason that analysis realized for SNP by ethnicity is more comprehensive, we choose its merger result as the overall result” in Page 11 in manuscript.
30. Page 13, second paragraph, “rs3825942” should be “rs2165241”.

**Reply:** We have revised the mistake in Page 14 in manuscript.
31. Page 13, second paragraph, it should be clarify in the text that the data “T vs. C, OR: 1.61, 95%CI: 1.18-2.19, p:0.002” are shown in Fig5.

**Reply:** We have clarified the question in Page 14 in manuscript.
32. Page 13, second paragraph, “Fig4” should be “Fig4, Table 2”.

**Reply:** We have added the Table2 in Page 14 in manuscript.
33. Page 14, first paragraph, “Fig5” should be “Fig5, Table 2”.

**Reply:** We have added the Table2 in Page 14 in manuscript.
34. Page 14, second paragraph, it should be clarify in the text that the data “G vs. A, OR: 5.33, 95%CI: 3.49-8.16, p<0.001” are shown in Fig7.

**Reply:** We have clarified the question in Page 14 in manuscript.
35. Page 14, second paragraph, “Fig6” should be “Fig6, Table 2” and “Fig7” should be “Fig7, Table 2”

**Reply:** We have added the Table2 in Page 14 in manuscript.
36. Page 14, second paragraph, the dot must be replaced by a comma. “…increased risks were identified among Caucasians (G vs. A, OR: 6.48, 95%CI: 3.67-11.44, P<0.001) and Asians (G vs. A, OR: 5.89, 95%CI: 3.79-9.16, p <0.001) (Fig 7), suggesting that…”

**Reply:** We have revised the dot to comma in the sentence in Page 15 in manuscript.
37. Figures 2-7 legends: The reader should be provided with more details about the figures. For instance, what the squares (or the diamonds) and their size indicate.

**Reply:** Squares depict individual studies and diamonds depict summary effect size estimates (Odds Ratio, OR).

38. Figures 2-7 legends: Please, replace “LOXL1 gene polymorphism in rs…” by “LOXL1 gene polymorphism rs…”

**Reply:** “LOXL1 gene polymorphism in rs…”is replaced by “LOXL1 gene polymorphism rs…”
39. Figure 8: Please, correct the leyend: Begg’s funnel plot for evaluation of publication bias in the selection of studies on the association between between exfoliation syndrome /exfoliation glaucoma risks and LOXL1 gene polymorphism, rs1048661(A), rs2165241(B) and rs3825942(C).

**Reply:** It could be corrected “Figure 8: (A)Begg’s funnel plot of publication bias for LOXL1 gene polymorphism rs1048661; (B) Begg’s funnel plot of publication bias for LOXL1 gene polymorphism rs2165241; (C) Begg’s funnel plot for of publication bias for LOXL1 gene polymorphism rs3825942”.
40. Page 15, Discussion: The statement “Various population studies have provided inconclusive evidence that LOXL1 single nucleotide polymorphisms are strongly
associated with XFS/XFG” should be qualified.
As mentioned in point 18, despite the existence of discrepancies between some studies related with the risk alleles of LOXL1 SNPs, it is widely accepted that LOXL1 gene is the most important genetic risk factor known so far for XFS/XFG.
Moreover, references 6, 70, 71 are not suitable.

**Reply:** We have deleted the statement “Various population studies have provided inconclusive evidence that LOXL1 single nucleotide polymorphisms are strongly
associated with XFS/XFG” in Page 16 in manuscript and revised the references.

41. Page 15, Discussion: Please, remove the reference 3 which has been retracted by the editor of International Journal of Ophthalmology.

**Reply:** We have removed the reference 3 which has been retracted by International Journal of Ophthalmology in Page 2 in manuscript.
42. Page 16, line 4: The number of participants was 36658 (cases + controls)

Reply: There is a mistake about the number, and we have revised it in Page 18 in manuscript..
43. Page 16: Risk alleles should be specified in the sentence “XFS/XFG analysis by ethnicity revealed significantly high association between the 3 LOXL1 polymorphisms and XFS/XFG risk in Caucasians”.
“XFS/XFG analysis by ethnicity revealed significantly high association between the allele G of rs1048661, the allele T of rs2165241 and the allele G of rs3825942 and XFS/XFG risk in Caucasians”

Reply: The sentence has been revised in Page 18 in manuscript.
44. The same should be done in the rest of the paragraph with all SNPs, since alleles that confer risk vary between ethnic groups. The risk alleles should be specified. For instance: “…the variant G of rs1048661 polymorphism may have potentially negative effects on…”
Moreover, some mistakes should be corrected: For instance: “In Asians, significantly increased XFS/XFG risk was associated with the two LOXL1 polymorphisms (rs3825942)” should be “In Asians, significantly increased XFS/XFG risk was associated with the allele G of rs3825942”.
Please, revise, correct and rewrite the paragraph in a more orderly way.

**Reply:** We have revised the sentence in Page 18 in manuscript.
45. The lack of studies in Africans, specially about the SNP rs2165241 should be mentioned in the text.

**Reply:** I have mentioned the lack of studies in Africans, specially about the SNP rs2165241 in Page 19 in manuscript.
46. In my opinion, mention should be made of the high frequencies of risk alleles in the controls and discuss their implications.

**Reply:** I have mentioned the high frequencies of risk alleles in the controls and discuss their implications in Page 19 in manuscript.

47. Page 16, Discussion: Differences and similarities with the results of other studies or with previous meta-analysis must be described and discussed in the manuscript. Does this work make any new contribution to the field or confirm the results of previous studies?

**Reply:** I have discussed differences and similarities with the results of other studies or with previous meta-analysis in Page 17 in manuscript.
48. Page 16: Clarify the sentence: “…the differences in genetic susceptibility might be affected by ethnic factors and periods of glaucoma”.
Ethnic factors can affect genetic susceptibility, the genetic background is different between ethnicities. However, the stage of glaucoma can not affect genetic susceptibility. The genotype of an individual do not change, is the same throughout his life regardless of the stage of a disease

**Reply:** “…the differences in genetic susceptibility might be affected by ethnic factors and periods of glaucoma” should be revised to “…the differences in genetic susceptibility might be affected by ethnic factors” in Page 19 in manuscript.
49. Page 17: Please, provide details of the studies that could support the hypothesis: “We hypothesized that the LOXL1 gene polymorphisms may upregulate LOXL1 production and that the high serum concentration of LOXL1 may contribute to XFS/XFG susceptibility”.

**Reply:** We added two references, [76] and [77].
50. Page 17: Please, correct the sentence“… hence the observed opposite rs1048661 risks in Asian vs Caucasians subjects”.
The effect of rs2165241 is also opposite in Asian and Caucasian

**Reply:** it is corrected to “the effect of rs2165241 and rs1048661 are opposite in Asian and Caucasian” in Page 19 in manuscript.
51. Page 17: Please, rewrite the sentence: “Heterogeneity involved in studies is generally in meta-analysis for the quality of the included research, population characteristics, experimental methods and other reasons”.

**Reply:** Delete this sentence: “Heterogeneity involved in studies is generally in meta-analysis for the quality of the included research, population characteristics, experimental methods and other reasons” .
52. Page 17: Please, correct or clarify the sentence: “Thus, we speculated that there are several other factors leading to heterogeneity: Blood sample sampling and storage methods are different; the measurement method and experimental conditions are different; varying levels of illness severity”.
In my opinion the factors leading to heterogeneity listed by the authors are irrelevant to a genetic study. The genotype of an individual is not affected by factors such as blood sample sampling or storage method. The mentioned factors could be relevant for other types of studies, for instance when quantification of a protein in blood is required.

**Reply:** Thus, we speculated that living environment and other complications might lead to heterogeneity in Page 20 in manuscript.
