# Supplementary material for: LOXL1 gene polymorphisms are associated with exfoliation syndrome/exfoliation glaucoma risk: An updated meta-analysis
Source: PLoS One. 2021 Apr 28;16(4):e0250772. doi: 10.1371/journal.pone.0250772 (PMC8081202; doi:10.1371/journal.pone.0250772)
Supplement: Attachment — Submitted filename: Response to Reviewers.doc [file pone.0250772.s003.doc]

**Reply:** The authors would like to thank the Reviewers and the Editor for their time in evaluating our study and for making useful suggestions. We believe that we have addressed all of the comments below, but we are open to any additional ones.

**Reply to Editors：**

1. Any changes to the reference list should be mentioned in the rebuttal letter that accompanies your revised manuscript.

**Reply**: We apologize for the mistake in quoting the two references [75,76] because we misunderstand the two references’ meaning. So, we removed these two references.

Discussion, pag 20: “Based on previous studies and the findings from the present meta-analysis, we hypothesized that the LOXL1 gene polymorphisms may upregulate LOXL1 production and that the high serum concentration of LOXL1 may contribute to XFS/XFG susceptibility [75,76]”. Neither the present study nor the references provided by the authors support this hypothesis.The works of Greene et al. (75) and Want et al. (76) have not studied the relationship between LOXL1 variants and dysregulation of LOXL1 production. According to Greene et al., (75) LOXL1 expression is downregulated in XFG due to DNA methylation and LOXL1 promoter methylation, while Want et al. (76) associate XFS with dysfunction in autophagy. In these studies, there is no mention of LOXL1 variants. On the other hand, the present study has not analyzed the production of LOXL1 and its relationship with LOXL1 variants.No indication has been provided to support that LOXL1 gene variants may upregulate LOXL1 production.

**Reply to Reviewers：**

**Reply:** We thank the reviewers’ comments and excellent suggestions. We have revised these mistakes and inaccuracies in the text as you pointed out, rewritten some references and revised some parts of the text. Reply to comments and suggestions of reviewers as follows:

1. Pag 1, Abstract Background, line 1: LOXL1 (do not use italics when referring to protein)

**Reply:** We have corrected the text of *LOXL1*.
2. Pag 1, Abstract Results: The number of controls in the sentence “In total, 5022 cases and 10400 controls were included in this meta-analysis” should be corrected.Table 1 show the number of XFS and XFG cases and controls in each study. Some studies report the results for XFS and XFG separately, in these studies the control group is the same for both diseases, but it was counted in duplicate. For example, in the case of the study of Panday et al., there is only a group control with 61 individuals.

**Reply:** In total, 5022 cases and 8962 controls were included in this meta-analysis. The total number of participants has been corrected in discussion too

3. Pag 2, Abstract results: Delete the word “only” in the sentence: “In addition, only rs1048661 and rs3825942 correlated with XFS/XFG susceptibility in Africans.The SNP rs2165241 was not analyzed in Africans. Authors do not know if this SNP is associated with XFS/XFG.

**Reply:** We have deleted the word “only” in the sentence.

4. Pag 3, Introduction: in the sentence “…as well as in most studies carried out in Caucasians [16,17]” delete reference 17, and add references of other studies in Caucasians

**Reply:** We have replaced the reference 17 to the reference 13

5. Pag 3-4, Introduction: Delete the word “where” in the sentence: “However, in most studies in Asians, where the alleles T and C of rs1048661 and rs2165241, respectively, are the risk alleles”.

**Reply:** We have deleted the word “where” in the sentence

6. Pag 4, Introduction: Please, change the sentence: “However, no significant association between XFS/XFG and SNP rs1048661 was observed” to “However, in contrast to what was observed in most Caucasian populations, no significant association between XFS/XFG and SNP rs1048661 was observed”.

**Reply:** We have changed the sentence: “However, no significant association between XFS/XFG and SNP rs1048661 was observed” to “However, in contrast to what was observed in most Caucasian populations, no significant association between XFS/XFG and SNP rs1048661 was observed”.

7. Page 6, Study characteristics: Figure 1 has not been corrected according to the text.

Original figure 1 has been included in the revised manuscript

“Our initial literature search returned 197 articles. Upon browsing the titles and abstracts, 111 articles were excluded, leaving 86 articles that underwent full-text review”.

Titles and abstracts were browsed in the first step, not in the second as is shown in the figure 1.

**Reply:** The 197 articles were retrieved without screening**, s**o, titles and abstracts were browsed in the second step.

8. Please, review the number of records excluded because they involved other LOXL1 gene polymorphisms. 32 in the text and 31 in the figure 1.

Review also the numbers in the text: “Although five articles [10,30-33] had been analyzed in a previous meta-analysis [34], we excluded them because three articles [10,30,31] did not achieve HWE in the control group, and two articles [32-33] reported the relationship between LOXL1 gene polymorphisms and primary open-angle glaucoma”. The numbers in the text must match those in the figure 1.

**Reply:** There are some vague descriptions in the article, and the numbers in the text are match those in Figure 1 indeed. So, we have revised some parts of the text to match with the Fig 1.

9. Figure 5 appears in the text before figure 4.

**Reply:** We have revised it.

10. Figure 7 appears in the text before figure 6.

**Reply:** We have revised it.

11. Discussion, pag 20: “Based on previous studies and the findings from the present meta-analysis, we hypothesized that the LOXL1 gene polymorphisms may upregulate LOXL1 production and that the high serum concentration of LOXL1 may contribute to XFS/XFG susceptibility [75,76]”.

Neither the present study nor the references provided by the authors support this hypothesis.

The works of Greene et al. (75) and Want et al. (76) have not studied the relationship between LOXL1 variants and dysregulation of LOXL1 production. According to Greene et al., (75) LOXL1 expression is downregulated in XFG due to DNA methylation and LOXL1 promoter methylation, while Want et al. (76) associate XFS with dysfunction in autophagy. In these studies there is no mention of LOXL1 variants

On the other hand, the present study has not analyzed the production of LOXL1 and its relationship with LOXL1 variants.

No indication has been provided to support that LOXL1 gene variants may upregulate LOXL1 production.

**Reply**: We apologize for the mistake in quoting the two references [75,76] because we misunderstand the two references’ meaning. So, we removed these two references.

Sharma et al. [59] reported that the coding variants rs1048861 and rs3825942 may alter protein function and binding, wherein molecular modeling displayed that positions 141(rs1048661) and 153(rs3825942) of the LOXL1 protein are likely surface residues and hence possible recognition sites for protein-protein interactions. Alterations at these residues might change the capacity of LOXL1 to bind other proteins related to its cleavage as well as processing. Nevertheless, the difference in processing of the LOXL1 protein variants detected in their research does not completely interpret susceptibility to XFS/XFG among carriers of these variants as each of the variants confer the XFS/XFG risk in various ethnicities. The detailed mechanism whereby *LOXL1* gene polymorphisms lead to the XFS/XFG, remains poorly understood. Therefore, Further studies are required to elucidate the mechanism on how the *LOXL1* gene polymorphisms impact the occurrence and development of XFS/XFG.
