# Supplementary material for: LOXL1 gene polymorphisms are associated with exfoliation syndrome/exfoliation glaucoma risk: An updated meta-analysis
Source: PLoS One. 2021 Apr 28;16(4):e0250772. doi: 10.1371/journal.pone.0250772 (PMC8081202; doi:10.1371/journal.pone.0250772)
Supplement: Attachment — Submitted filename: 4.8-Response to Reviewers.doc [file pone.0250772.s004.doc]

**Reply:** The authors would like to thank the Reviewers and the Editor for their time in evaluating our study and for making useful suggestions. We believe that we have addressed all of the comments below, but we are open to any additional ones.

**Reply to Reviewers：**

**Reply:** We thank the reviewers’ comments and excellent suggestions. We have revised these mistakes as you pointed out. Reply to comments and suggestions of reviewers as follows:

1. Abstract, Background: “… in the gene encoding the LOXL1…” should be “… in the gene encoding LOXL1…”

**Reply:** We have revised the mistake in page 1.

2. Discussion, Pag 18: “… the allele T of rs2165241 had a potential protective effect on XFS/XFGS in Asians.” XFG not XFGS

**Reply:** We have revised the mistake in page 18.

3. Discussion, Pag 19: “However, we also found that LOXL1 the G allele of rs1048661..." should be “However, we also found that the G allele of rs1048661 ..."

**Reply:** We have revised the mistake in page 19.

4.Discussion, Pag 19: “Additionally, there was a significant association between LOXL1 gene polymorphisms and susceptibility to various disease types. These results affirmed the association between LOXL1 gene polymorphisms and XFS and XFG.” LOXL1 in italics when referring to the gene

**Reply:** We have corrected the text of *LOXL1* in page 19.
